# Phase I/II trial of cilengitide with cetuximab, cisplatin and 5-fluorouracil in recurrent and/or metastatic squamous cell cancer of the head and neck: findings of the phase I part

**DOI:** 10.1038/bjc.2011.152

**Published:** 2011-05-03

**Authors:** J B Vermorken, J Guigay, R Mesia, J M Trigo, U Keilholz, A Kerber, U Bethe, M Picard, T H Brummendorf

**Affiliations:** 1 Department of Medical Oncology, Antwerp University Hospital, Wilrijkstraat 10, Edegem 2650, Belgium; 2 Department of Medicine, Head and Neck and Thoracic Medical Oncology, Gustave Roussy Institute, 39 rue Camille Desmoulins, Villejuif 94805, France; 3 Department of Medical Oncology, Catalan Institut of Oncology – L’Hospitalet, Barcelona 08097, Spain; 4 Department of Oncology, Day Hospital, Malaga 29010, Spain; 5 Hematology and Medical Oncology, Benjamin Franklin University, Medical Clinic III, Hindenburgdamm 30, Berlin 12200, Germany; 6 Merck KGaA, Frankfurter Str. 250, Darmstadt 64293, Germany; 7 Department of Oncology and Hematology, University Hospital Hamburg-Eppendorf, Martinistr. 52, Hamburg 20246, Germany; 8 Department of Hematology and Oncology, University Hospital Aachen, Pauwelsstrasse 30, Aachen 52074, Germany

**Keywords:** cilengitide, SCCHN, cetuximab, cisplatin, 5-FU, safety

## Abstract

**Background:**

Novel therapies are needed to improve the poor prognosis of patients with recurrent and/or metastatic squamous cell cancer of the head and neck (SCCHN).

**Methods:**

ADVANTAGE is a phase I/II, multicentre study evaluating the integrin inhibitor cilengitide combined with cetuximab and platinum-based chemotherapy in patients with recurrent and/or metastatic SCCHN. The phase I part tested cilengitide (500, 1000 and 2000 mg) twice weekly with standard doses of cetuximab, cisplatin and 5-fluorouracil.

**Results:**

Ten patients (9 male, 1 female; median 56 years old) were included in the phase I part. No dose-limiting toxicities (DLTs: grade 3/4 toxicities in the first 3 weeks as defined per protocol) or deaths occurred. The most common adverse events (AEs) were constipation, rash, nausea, anorexia and fatigue. Cilengitide-related grade 3/4 AEs, all of which occurred after the DLT observation period, were anaemia, angioedema, asthenia, mucosal inflammation, nausea and vomiting (one event per category). Best overall tumour response was partial response (PR) for 4 out of 10 patients and stable disease (SD) for 6 out of 10 patients across all cohorts. Disease control rate (complete response, PR and SD) was 100%.

**Conclusion:**

Cilengitide combined with cetuximab and platinum-based chemotherapy was well tolerated. No DLTs or unexpected AEs were observed. Cilengitide 2000 mg was considered safe and was selected for the subsequent randomised phase II part assessing progression-free survival.

Most cancers of the head and neck are located in the oral cavity, pharynx and larynx (National Comprehensive Cancer Network, 2010), and >90% of such cancers are of squamous cell histology (Marur and Forastiere, 2008; Gregoire *et al*, 2010). Squamous cell cancer of the head and neck (SCCHN) accounted for 4.0% of all cancers worldwide in 2008, with an estimated 498 000 new cases globally in that year (Ferlay *et al*, 2010).

The prognosis for patients with recurrent and/or metastatic disease is extremely poor, and has remained largely unchanged in the past 30 years despite the introduction of new cytotoxic agents. The treatment goal for recurrent and/or metastatic SCCHN is symptom management and prolongation of survival (National Comprehensive Cancer Network, 2010). For those not suitable for local therapies, treatment options have been limited to systemic chemotherapy and best supportive care (Langer, 2008). With the use of platinum-based chemotherapy, median survival time is typically around 6–8 months (Langer, 2008) and the 1-year survival rate is 20–40% (Gibson *et al*, 2005).

The introduction of therapies targeting the epidermal growth factor receptor (EGFR) has recently improved prognosis in SCCHN patients. In a large randomised controlled trial published in 2008, the addition of cetuximab, a monoclonal antibody against EGFR, to platinum-based chemotherapy was associated with a significant improvement in overall survival (OS) and progression-free survival (PFS) and good tolerability, compared with chemotherapy alone (Vermorken *et al*, 2008). Median OS was 10.1 months (*vs* 7.4 months with chemotherapy alone, *P*=0.04) and median PFS was 5.6 months (*vs* 3.3 months with chemotherapy alone, *P*<0.001) (Vermorken *et al*, 2008). This increase in OS time of 2.7 months was a significant advance in the treatment of SCCHN, but novel approaches are urgently required to further improve survival (Bernier, 2009).

Integrins promote and regulate endothelial cell proliferation, migration, invasion, and survival in tumours, securing vascularisation and vascular remodelling in tumours (Garmy-Susini and Varner, 2008; Desgrosellier and Cheresh, 2010). Squamous cell cancer of the head and neck is a highly vascularised cancer that expresses integrins: integrin *α*v*β*3 is primarily expressed in the endothelia of SCCHN tumours (Beer *et al*, 2007; Fabricius *et al*, 2011) while integrin *α*v*β*5 is primarily expressed in the tumour stroma (Fabricius *et al*, 2011). Cilengitide (EMD 121974, manufactured by Merck KGaA, Darmstadt, Germany) is an investigational cyclic arginine-glycine-aspartic acid (RGD) containing pentapeptide sequence that selectively inhibits the *α*v*β*3/5 integrins (Dechantsreiter *et al*, 1999). Cilengitide is the first integrin inhibitor to reach phase III clinical trials in glioblastoma, another highly vascularised cancer (Stupp *et al*, 2010b).

ADVANTAGE is a phase I/II trial evaluating cilengitide in combination with cetuximab, cisplatin and 5-fluorouracil (5-FU) in patients with recurrent and/or metastatic SCCHN. We report here the results of the phase I part of the ADVANTAGE study, which was designed to determine the safety and tolerability of the combination treatment at increasing doses of cilengitide.

## Materials and methods

### Study design

The ADVANTAGE trial (ClinicalTrials.gov identifier NCT00705016) is a phase I/II, multicentre study in patients with recurrent and/or metastatic SCCHN. The study is divided into two parts: a phase I part with dose escalation of cilengitide (presented here) and an open-label, randomised, controlled, phase II part, which is ongoing. The phase I part aimed to include 9–18 patients, with 3 patients per cohort, and began in July 2008. The data cutoff point for the safety results presented here was 1 March 2010.

A 3+3 design was used, consisting of a cohort-wise escalation of cilengitide in combination with cetuximab, cisplatin and 5-FU over three cohorts. Patients were observed for dose-limiting toxicities (DLTs) during the first 3-week cycle of therapy. If there were no DLTs in the three patients in cohort 1, then cohort 2 could proceed, but if one of the three patients from cohort 1 experienced a DLT, then an additional three patients were required to be evaluated in cohort 1. If a cohort was expanded to six patients in this way, the next cohort could proceed if ⩽2 of 6 patients experienced a DLT.

### Patients

Inclusion criteria for the study included: (i) adults aged ⩾18 years; (ii) a histologically or cytologically confirmed diagnosis of recurrent and/or metastatic SCCHN that was not suitable for local therapy; (iii) patients with ⩾1 measurable lesion by either computerised tomography (CT) or magnetic resonance imaging (MRI); (iv) performance status ⩾70 on the Karnofsky performance status scale; and (v) 0–1 on the Eastern Cooperative Oncology Group scale.

Key exclusion criteria were (i) prior systemic chemotherapy (unless as part of multimodal treatment for locally advanced disease completed >6 months before study entry); (ii) surgery (excluding diagnostic biopsy) or irradiation ⩽4 weeks before study entry; (iii) nasopharyngeal carcinoma; (iv) active infection, uncontrolled hypertension, abnormal haematology or liver function; (v) pregnancy; (vi) other concomitant anticancer therapies or previous treatment with EGFR targeting therapy or signal transduction inhibitors; (vii) brain metastases; and (viii) known drug abuse.

The study was conducted in accordance with the Declaration of Helsinki and in compliance with Good Clinical Practice. The protocol was approved by institutional review boards, and all patients provided written informed consent.

### Study intervention

The selection of cilengitide regimens in this study was based on investigations to date in patients with various cancer diagnoses. These studies demonstrated that twice weekly cilengitide up to 2400 mg m^−2^ had antitumour effects and was well tolerated (Eskens *et al*, 2003; Nabors *et al*, 2007).

Patients were given one of three cilengitide doses (500, 1000, or 2000 mg) administered intravenously (i.v.) twice a week over 1 h, in combination with standard doses of cetuximab (administered i.v. at an initial dose of 400 mg m^−2^ over 2 h, followed by weekly doses of 250 mg m^−2^ over 1 h) and chemotherapy. Chemotherapy consisted of cisplatin (100 mg m^−2^ i.v. on day 1) plus 5-FU (1000 mg m^−2^ day^−1^ continuous infusion from day 1 to 4) during the first week of each 3-week cycle. Treatment was administered for up to 6 cycles (i.e., 18 weeks), until progressive disease (PD), death, unacceptable toxicity or withdrawal of consent. Patients without progression after six cycles of therapy continued maintenance treatment with cilengitide plus cetuximab only.

### Measured outcomes

The primary objective of the phase I safety run-in was to determine the safety and tolerability of cilengitide in combination with cetuximab, cisplatin and 5-FU. The study also examined activity in terms of response and disease control. Imaging studies, including CT scans or MRI of the head, neck and chest, were performed at baseline and every 6 weeks after randomisation until PD. Response was assessed according to RECIST 1.0 (Therasse *et al*, 2000). Disease control rate was defined as the proportion of patients whose best overall response was confirmed complete response (CR), partial response (PR) or stable disease (SD).

Safety was assessed by monitoring laboratory parameters, vital signs and adverse events (AEs) as graded according to the National Cancer Institute Common Terminology Criteria (NCI CTCAE v3.0). Treatment-related AEs were defined as AEs that the investigator deemed related to the investigational medicinal product or non-investigational medicinal product. Investigators assessed relatedness of an AE either as ‘related’ or as ‘not related’.

### Definition of DLTs

The observation period for DLTs was the first 3 weeks of study treatment. During that time, DLTs were defined as follows:

- Any grade 3/4 treatment-related toxicity confirmed by the Safety Monitoring Committee to be relevant. This explicitly included:
  - Any grade 4 haematological or non-haematological toxicity if not specifically excluded (see below).
  - Any treatment-related death observed within the first 3 weeks of therapy.
- Explicitly excluded from the DLT definition were the following toxicities that are expected from treatment with cisplatin, 5-FU or cetuximab:
  - Fatigue (lethargy, malaise and asthenia – even if considered related to cilengitide).
  - Alopecia.
  - Rash of grades 3 and 4.
  - Nausea or vomiting of grades 3 and 4.
  - Neutropenia for ⩽5 days and not associated with fever.
  - Isolated lymphocytopenia or thrombocytopenia grades 3 and 4 with no clinical correlate.
  - Diarrhoea grades 3 and 4 in the absence of supportive care.
  - Single laboratory values that were out of the normal range, without clinical correlate, unlikely to be treatment-related and spontaneously resolving within 7 days.
  - Any hypersensitivity reaction (as this is independent of dose).

## Results

In total, 10 patients (9 male, 1 female) with a median age of 56 years (range 32–74 years) with recurrent and/or metastatic SCCHN participated in the phase I part (Table 1). Three patients were included in cohorts 1 and 2, respectively. Four patients instead of three were included in cohort 3, as two participants were screened in parallel and a request to include both in the trial was accepted. The median treatment duration of cilengitide administration was 24.4 weeks (range 12–57 weeks), and all patients completed at least three treatment cycles (Table 2). One patient completed three treatment cycles, two patients completed four treatment cycles and one patient completed five treatment cycles. Six patients completed all six treatment cycles and four out of the six patients continued maintenance treatment with cilengitide and cetuximab. The reasons for study completion/discontinuation were PD (eight patients), an AE (one patient who had elevated creatinine levels) and the investigator's decision (one patient). Individual patient characteristics are described in Table 3.

### Safety and tolerability

All patients (*n*=10) experienced at least one AE during the study; however, the observed AEs were in line with the patients’ underlying disease and the known toxicities of cetuximab and the concomitant chemotherapies. No DLTs (for DLT definition, see *Measured outcomes* section) or deaths were reported for any patient in any cohort during the phase I component of this study.

The most common AEs of any toxicity grade were constipation, rash, nausea, anorexia and fatigue (Table 4). Seven patients experienced an AE assessed as related to cilengitide by the investigator (one in cohort 1, three in cohort 2 and three in cohort 3). Of the most common AEs reported (in >2 patients), those assessed as related to cilengitide were nausea, anorexia, asthenia, vomiting, mucosal inflammation and dry skin (Table 4).

Grade 3/4 AEs were experienced by all 10 patients, and these were related to cilengitide in 3 patients (two in cohort 2 and one in cohort 3). Neutropenia was the most common grade 3/4 AE, but this was not assessed as related to cilengitide treatment (Table 5). Anaemia, angioedema, asthenia, mucosal inflammation, nausea and vomiting were grade 3/4 AEs that occurred after the DLT observation period and were assessed as related to cilengitide treatment. The single case of angioedema (of the throat) occurred 10 months after the start of treatment and 2 weeks after the very last administration of cetuximab and cilengitide. The investigator assigned angioedema as related to both cetuximab and cilengitide. However, the day before occurrence of angioedema, bisoprolol had been started in this patient. While anaphylactoid/anaphylactic reactions have been reported with cetuximab (Merck Serono, 2010), and angioedema has been observed with agents that have antiangiogenic properties (Bayer, 2011), the *β*-blocker bisoprolol is a known cause of angioedema (Duramed Pharmaceuticals, 2011). Bisoprolol was stopped the day after onset of angioedema and angioedema resolved the same day. Only one grade 4 AE occurred (a decreased neutrophil count in cohort 3) and it was not assessed by the investigator as related to cilengitide treatment.

The maximum tolerated dose of cilengitide was not reached.

### Efficacy parameters

The median PFS with cilengitide in combination with cetuximab and platinum-based chemotherapy was 5.88 months (95% CI 2.96–10.15). Best overall tumour response summarised in Table 2 was PR for one and three patients in the 1000 and 2000 mg groups, respectively, and SD for three, two and one patients in the 500, 1000 and 2000 mg groups, respectively. Disease control rate (CR, PR and SD) was 100%.

## Discussion

This phase I part of a combined phase I/II trial defined cilengitide 2000 mg twice weekly as safe when given with cetuximab, cisplatin and 5-FU for recurrent and/or metastatic SCCHN. Observed AEs were in line with both the underlying malignant condition and the known toxicities of cetuximab and concomitant chemotherapy. The maximum tolerated cilengitide dose was not identified. No DLTs were observed.

The selection of cilengitide dose and escalation schedule was based on previous investigations in animal models and in clinical studies of patients with various types of cancers (Eskens *et al*, 2003). In nude mice bearing M21-L human melanoma xenografts, optimal inhibition of tumour growth occurred at 10–15 mg kg^−1^, corresponding to peak plasma concentrations of 10.9–12.7 mg ml^−1^. In a single-agent dose-escalation phase I study of 37 patients with metastatic solid tumours, pharmacokinetic analysis showed that these plasma concentrations (11–13 mg ml^−1^) were achieved in humans at a dose level of 120 mg m^−2^ (Eskens *et al*, 2003). Ten dose levels of cilengitide (30–1600 mg m^−2^ i.v. twice weekly) were examined in this study and cilengitide was associated with a good safety profile. The pharmacokinetic profile of cilengitide was independent of dose and no DLTs occurred (Eskens *et al*, 2003). A further phase I study in 20 patients with advanced solid tumours investigated two doses of cilengitide (600 and 1200 mg m^−2^) administered i.v. twice weekly for a median number of 20 infusions per patient (Hariharan *et al*, 2007). No DLTs were associated with cilengitide, and it was well tolerated.

Cilengitide has previously been examined in patients with refractory brain tumours, in a phase I study of 31 paediatric patients (⩽21 years old) who received cilengitide at doses of 120–2400 mg m^−2^ i.v. twice weekly, for up to 52 weeks (MacDonald *et al*, 2008). The results were similar to those of the current study, with no DLTs observed. The twice weekly dosage of 1800 mg m^−2^ was recommended for the phase II part of the trial (MacDonald *et al*, 2008). An open-label phase I study investigated the toxicity and maximum tolerated dose of cilengitide in 51 adults with recurrent malignant glioma (Nabors *et al*, 2007). Based on the data from previous studies, cilengitide was administered in eight different doses from 120 to 2400 mg m^−2^ i.v. twice weekly in cohorts containing six evaluable patients (Nabors *et al*, 2007). There was no evidence of any cilengitide-related acute toxicities and a maximum tolerated dose was not identified. Some DLTs occurred and AEs were mostly mild and the majority were not attributable to cilengitide. Cilengitide was well tolerated up to doses of 2400 mg m^−2^. Several phase II trials have investigated the effects of cilengitide (500 or 2000 mg twice weekly) in patients with newly diagnosed or recurrent glioblastoma (Gilbert *et al*, 2007; Reardon *et al*, 2008; Stupp *et al*, 2010a). These studies found that cilengitide was well tolerated, with promising antitumour activity and no significant reproducible toxicities. The higher dose of cilengitide (2000 mg) was favourable with respect to OS and PFS and was thus recommended for subsequent trials (Nabors *et al*, 2007; Reardon *et al*, 2008; Stupp *et al*, 2010a).

The rationale for combining cilengitide with cetuximab and platinum-based chemotherapy was based upon preclinical data illustrating cross talk between integrins and members of the EGFR family (Desgrosellier and Cheresh, 2010). Also, there is evidence that integrin inhibitors may be most effective when combined with chemotherapies (Abdollahi *et al*, 2005; Desgrosellier and Cheresh, 2010). Pharmacokinetic analyses are planned for the phase II part of this study, as well as pharmacodynamic and pharmacogenetic assessments.

Despite recent therapeutic advances such as cetuximab combined with platinum-based chemotherapy, which can extend survival in patients with recurrent and/or metastatic SCCHN, patients still have a poor prognosis (Vermorken *et al*, 2008). Cilengitide is an integrin inhibitor, with a different mode of action compared with the other therapeutic agents currently in clinical practice for the treatment of SCCHN. It is hypothesised that cilengitide will increase antitumour activity without compromising safety and tolerability. A case report describes a patient with a highly proliferative squamous cell carcinoma of the upper left jaw (Raguse *et al*, 2004). The patient received 600 mg m^−2^ cilengitide i.v. twice weekly in combination with 1000 mg m^−2^ gemcitabine twice weekly every 3 weeks for 5 months followed by maintenance therapy with 600 mg m^−2^ cilengitide i.v. twice weekly in a clinical setting (Raguse *et al*, 2004). The patient remained stable for 12 months and achieved a partial remission, demonstrating the clinical potential of cilengitide (Raguse *et al*, 2004).

The current findings of the phase I part of ADVANTAGE are promising since they demonstrate an acceptable tolerability profile of cilengitide and absence of DLTs in patients with recurrent and/or metastatic SCCHN. The randomised phase II part of this trial is currently investigating PFS in patients with SCCHN treated with two regimens of 2000 mg cilengitide (weekly *vs* twice weekly administration) combined with cetuximab, cisplatin and 5-FU, compared with cetuximab, cisplatin and 5-FU alone. On the basis of the available preclinical and clinical phase I/II data for cilengitide in recurrent malignant glioma, and the findings from this phase I part, two regimens have been selected for the phase II part of ADVANTAGE: cilengitide 500 mg four times a week (week 1) followed by 2000 mg cilengitide (weeks 2 and 3) for group A or 2000 mg cilengitide twice weekly for group B.

In conclusion, the current study investigates a combination of the integrin inhibitor cilengitide with cetuximab and platinum-based chemotherapy in patients with recurrent or metastatic SCCHN. In the phase I safety run-in, cilengitide in combination with cetuximab, cisplatin and 5-FU was well tolerated, associated with no unexpected AEs and no DLTs, and no maximum tolerated dose was identified. A dose of cilengitide 2000 mg was selected for the phase II study, which will assess PFS in a larger patient group.

## Figures and Tables

**Table 1:** Patient demographics

	**Cohort 1 (*n*=3)**	**Cohort 2 (*n*=3)**	**Cohort 3 (*n*=4)**
**Cilengitide**	**500 mg**	**1000 mg**	**2000 mg**
Median (range) age, years	56 (54–74)	43 (32–59)	57 (49–68)
Male	3	2	4
Female	–	1	–
ECOG performance status, *n*			
0	2	1	1
1	1	2	3

Abbreviation: ECOG=Eastern Cooperative Oncology Group.

**Table 2:** Cilengitide exposure and response

	**Cohort 1 (*n*=3)**	**Cohort 2 (*n*=3)**	**Cohort 3 (*n*=4)**
**Cilengitide dose**	**500 mg**	**1000 mg**	**2000 mg**
Median duration of cilengitide administration (range), weeks	23.3 (14–25)	42.4 (35–57)	16.3 (12–45)
			
*Best overall tumour response, n*			
Complete response	0	0	0
Partial response	0	1	3
Stable disease	3	2	1
Progressive disease	0	0	0

**Table 3:** Patient characteristics

**ID**	**Gender**	**Age (years)**	**Locoregional recurrence or metastasis of SCCHN**	**Localisation of metastases**	**Histological grading of squamous cell carcinoma**	**TNM** **stage at *first diagnosis* of SCCHN**	**Primary tumour site**	**ECOG performance status**
02010001	Male	56	Distant metastasis	Liver	Unknown	T3N2M0	Pharynx	0
02010002	Female	43	Locoregional recurrence	Not applicable	Well differentiated	T1N0M0	Tongue	1
02010003	Male	56	Distant metastasis	Liver	Moderately differentiated	T4N2M1	Pharynx	0
03010001	Male	59	Locoregional recurrence	Not applicable	Unknown	TxNxMx	Tongue	1
03010002	Male	68	Locoregional recurrence	Not applicable	Well differentiated	T4N1M0	Pharynx	1
04010001	Male	54	Locoregional recurrence	Not applicable	Moderately differentiated	T4N1M0	Larynx	0
04010002	Male	74	Distant metastasis	Lung	Poorly differentiated	T4N3M1	Pharynx	1
04010004	Male	49	Distant metastasis	Cutaneous, skeletal, lymph nodes	Poorly differentiated	T4N3M0	Larynx	1
08010001	Male	32	Distant metastasis	Lung, hepatic, kidney	Poorly differentiated	T1N1M1	Tongue	0
08010002	Male	57	Locoregional recurrence	Not applicable	Well differentiated	T2N2M0	Tongue	1

Abbreviations: ECOG=Eastern Cooperative Oncology Group; SCCHN=squamous cell cancer of the head and neck; TNM = tumour node metastasis.

**Table 4:** Most frequent AEs (reported in >2 out of 10 patients) and most frequent AEs assessed as related to cilengitide (>2 out of 10 patients in total)

	**Most frequent AEs^a^^,^^b^**	**Most frequent AEs related**^c^ **to cilengitide (*n*=10 patients)**^a^		
	***n*=10 patients**	**Cohort 1 (*n*=3)**	**Cohort 2 (*n*=3)**	**Cohort 3 (*n*=4)**
**Cilengitide**	**All doses**	**500 mg**	**1000 mg**	**2000 mg**
Constipation	8			
Rash	7			
Nausea	7	0	3	1
Anorexia	7	1	2	1
Fatigue	6			
Asthenia	5	0	2	2
Pyrexia	5			
Diarrhoea	5			
Vomiting	5	0	2	1
Skin fissures	4			
Stomatitis	4			
Anaemia	4			
Neutropenia	4			
Mucosal inflammation	3	0	1	2
Dry skin	3	0	2	1
Hypokalaemia	3			
Dizziness	3			
Paraesthesia	3			

Abbreviation: AEs=adverse events.

a Regardless of toxicity grade.

b Any AEs, regardless of relatedness to cilengitide.

c Investigators assessed AEs either as ‘related’ or as ‘not related’.

**Table 5:** Grade 3/4 AEs and their relatedness to cilengitide

	**Cohort 1 (*n*=3)**	**Cohort 2 (*n*=3)**	**Cohort 3 (*n*=4)**
**Cilengitide**	**500 mg**	**1000 mg**	**2000 mg**
*Grade 3/4 AEs*			
Anaemia	2		1^a^
Angioedema		1^a^^,^^b^	
Asthenia		1^a^	
Deafness	1		
Decreased neutrophil count			1
Decreased white blood cell count			1
Dehydration			1
Diarrhoea			1
Dizziness			1
Fatigue	1	1	
Febrile neutropenia	1		
Hypertension		1	
Hypocalcaemia	1		
Hypokalaemia	1		1
Hypophosphataemia		1	
Lung infection	1		
Mucosal inflammation		1^a^	1
Nausea		1^a^	
Neutropenia	1	1	2
Pneumonia		1	
Vomiting		1^a^	1

Abbreviation: AEs=adverse events.

a Assessed as related to cilengitide: all of these occurred after the DLT observation period.

b See more detailed information on angioedema in *Results* section.
